# Central Nervous System Fungal Infections in Pakistan: A Narrative Review and Descriptive Synthesis

**DOI:** 10.7759/cureus.111614

**Published:** 2026-06-27

**Authors:** Fiza Ismail, Farah Ismail, Joshua Jamil, Abdul Rehman, Maazan Mubasher, Syed Haider Hassan, Sameed Safdar, Muhammad Fawad Ul Hassan, Faiqa I Khan, Arham Amir Khawaja, Haseeb Mehmood Qadri

**Affiliations:** 1 Medicine, Continental Medical College, Lahore, PAK; 2 Medicine, Rawalpindi Medical University, Rawalpindi, PAK; 3 Clinical Research, Shalamar Medical and Dental College, Lahore, PAK; 4 Medicine, Lahore Medical and Dental College, Lahore, PAK; 5 Medicine, Nishtar Medical University, Multan, PAK; 6 Medicine, Allama Iqbal Medical College, Lahore, PAK; 7 Neurological Surgery, National Hospital and Medical Centre, Lahore, PAK; 8 Neurological Surgery, Punjab Institute of Neurosciences, Lahore, PAK; 9 General Surgery and Surgical Oncology, Shaikh Zayed Medical Complex, Lahore, Lahore, PAK; 10 General Surgery, Lahore General Hospital, Lahore, PAK

**Keywords:** aspergillosis, brain diseases, central nervous system, cerebral abscess, fungal, meningitis, fungal infections

## Abstract

Central nervous system (CNS) fungal infections are associated with significant morbidity and mortality globally, especially in immunocompromised patients. This narrative review aimed to characterise the spectrum of CNS fungal infections in Pakistan. Overall, 14 studies with histologically confirmed diagnoses of CNS fungal infection were included, comprising data on 140 patients. The average age was 38.7 years with male predominance. The most common presenting symptom was headache in 58 (41.42%), followed by visual disturbance in 27 (19.28%) and limb weakness in 22 (15.71%). Among all patients, 31 (22.14%) had a history of diabetes mellitus. *Aspergillus *was the most frequently isolated pathogen in 46 (32.85%) patients, followed by *Mucorales *in 42 (30%) patients. Combined medical and surgical management was used in 113 (80.71%) cases. The primary antifungal treatment administered was amphotericin B and voriconazole in 82 (58.57%) and 41 (29.28%) patients, respectively. The surgical management included neuronavigation-guided biopsy in 29 (20.71%), endoscopic debridement in 16 (11.42%), and subtotal resection in 15 (10.71%). The combined medical and surgical management resulted in clinical improvement in 57 (50.44%) patients and mortality in 41 (36.28%) patients. Among the patients given only medical therapy, mortality occurred in 17 (65.38%) patients, and resolution occurred in 2 (7.69%) patients.

This review demonstrates that CNS fungal infections in Pakistan often present with nonspecific symptoms. Diabetes mellitus was the most commonly reported comorbidity. The management is multimodal, with combined medical and surgical management required in the majority of patients. Despite treatment, mortality remains high, emphasising the need for earlier diagnosis.

## Introduction and background

Central nervous system (CNS) fungal infections are rare but present with potentially life-threatening conditions associated with substantial morbidity and mortality [1]. They usually manifest as meningitis, encephalitis, hydrocephalus, stroke, and cerebral abscess [1]. The most commonly reported fungal species include *Aspergillus*, *Cryptococcus*, *Rhinocladiella*, *Mucorales*, and *Candida *[2]. These fungi usually enter the human body through the respiratory tract and spread to the CNS via the bloodstream [3]. Once fungi enter the bloodstream, they are capable of initiating a strong immune response depending on the virulence of the infective organism and the immunity of the human host [3,4]. Moreover, recent advances in medical therapy and environmental determinants have also led to increased susceptibility and the emergence of drug-resistant pathogens [2]. The diagnosis of CNS fungal infection remains limited due to the similarity in presentation with other endemic intracranial pathologies. Furthermore, the limited access to antifungal therapy contributes to disproportionately high morbidity and mortality rates in low- and middle-income countries [5].

Pakistan has several high-risk factors for CNS fungal infections, including significantly high rates of diabetes, a high prevalence of tuberculosis (TB), human immunodeficiency virus (HIV), chronic diseases, and increased usage of immunosuppressive medications due to autoimmune diseases [6,7]. The overlapping symptoms with TB meningitis, restricted neuroimaging availability, and low clinical and public awareness all contribute to the underdiagnosis and underreporting of CNS fungal infections [8]. Despite the high prevalence of predisposing conditions such as diabetes mellitus, tuberculosis, and use of immunosuppressive therapy in Pakistan, the evidence regarding CNS fungal infection remains fragmented and largely limited to isolated case reports and small institutional studies. Consequently, a consolidated evidence base to guide diagnosis, management, and future research priorities in the regional context and resource-limited setting remains scarce. Furthermore, this narrative review aims to integrate regional data to identify differences in regional and global evidence on the burden, highlight diagnostic obstacles in resource-constrained settings, and optimise the tailored management for the region.

## Review

Methodology

This narrative review consolidates the evidence of CNS fungal infections in Pakistan, using PubMed and Google Scholar. Given that the published literature primarily consisted of isolated case reports and small case series with substantial heterogeneity in clinical presentation, patient characteristics, diagnostic approaches, and outcome reporting, a PRISMA-guided systematic review was not feasible. Therefore, a narrative review approach was adopted to provide a comprehensive overview of the available evidence. The extracted studies were screened manually according to their title and predefined eligibility criteria.

Eligibility criteria

Case reports, case series, and original articles with pathologically confirmed CNS fungal infections from 2010 to 2024 were included. Included studies required to be documented in English and involve human participants. Opinion pieces, commentaries, conference abstracts, animal studies, and cadaveric studies were excluded. Studies were screened, and only those focusing on fungal infections of the brain were selected, while those involving the spinal cord were excluded.

Search strategy and data extraction

The primary data search was conducted by authors F.I.K. and F. Ismail to extract studies according to predefined keywords and Boolean operators. In case of discrepancies, author H.M.Q. was consulted. The combinations used were as follows: ‘fungal brain abscess Pakistan’, ‘fungal infections brain Pakistan’, ‘fungal CNS Pakistan’, ‘aspergillosis CNS Pakistan’, ‘aspergillosis brain’ AND Pakistan’, ‘neurofungal infections Pakistan’, ‘brain fungal infections Pakistan’, ‘fungal meningitis Pakistan’, ‘fungal myelitis Pakistan’, ‘aspergillosis CNS Pakistan’, ‘cerebral aspergillosis Pakistan’, ‘cryptococcal meningitis Pakistan’, ‘mucormycosis CNS Pakistan’, ‘candida CNS infection Pakistan’, ‘aspergillus CNS Pakistan’, ‘mucormycosis CNS Pakistan’, ‘rhizopus CNS Pakistan’, ‘neuroinvasive fungal infection’ AND ‘Pakistan’. The studies were identified from databases and search engines, namely, PubMed and Google Scholar. A total of 188 articles were identified using the predefined keywords from PubMed, and about 15,100 from Google Scholar. Four independent data extractors (M.M., S.S., J.J., A.R.) manually screened the data from the databases; after excluding duplications and undertaking the defined eligibility criteria, 14 studies with a pathologically confirmed diagnosis of human CNS fungal infection were included, containing data of 140 patients (Table 1).

**Table 1:** Search strategy employed to find Pakistani studies related to central nervous system fungal infections

Search strategy	Number of publications
Total publications identified using PubMed and Google Scholar databases	15,288
Total number of duplicate publications removed after screening	10,420
Publications screened based on title relevance	4,868
Publications excluded due to irrelevance	4,200
Publications screened based on title and abstract	668
Publications excluded after the title or abstract screening	620
Full-text articles assessed for eligibility	48
Total studies included in the review	14

Data stratification and analysis

Both quantitative and qualitative variables were extracted, and the data were recorded in the pre-made tabulations on Microsoft Word 365 (Microsoft Corp., Redmond, USA) incorporating the following variables: patients' demographic details and study type, symptomatology, physical and neurological examination findings, identifiable risk factors, past medical history, spectrum of fungal organisms identified, distribution of brain region involvement, management (medical and surgical details), and outcome-related parameters. The variables were analysed as a quantitative assessment, and descriptive statistics were used. Data were analysed using IBM SPSS Statistics for Windows, version 24 (IBM Corp., Armonk, USA), and descriptive tabulations were generated. The details of the included studies are listed in Table 2.

**Table 2:** Review of the literature documenting CNS fungal infections managed neurosurgically in Pakistani population

Author	Study title	Study type	Age/gender (M/F)	Number of patients
Nawaz‎ et al. [5]	Fungal infections of ‎the central nervous ‎system in the ‎seemingly ‎immunocompetent - ‎common or unusual	Cohort study	‎34.88 ± 19.49‎	21; M, 17 (81.0%);‎ F, 4 (19.0%)
Ikram et al. [6]	Rhinocerebral zygomycosis in Pakistan: clinical spectrum, management, and outcome	Cohort study	40 ± 5.0	36; M, 23 (63.8%); F, 13 (36.1%)
Bhatti et al. [7]	An unusual presentation of fungal brain abscess in immunocompetent host	Case report	30/F	1
Ali et al. [8]	Cladophialophora bantiana as a cause of rare fungal brain abscess	Case series	23/M, 27/M	2
Mir et al. [9]	Madurella mycetomatis as an agent of brain abscess: case report and review of literature	Case report	7/M	1
Khan et al. [10]	A unique MRI presentation of fungal infection in the brain	Case report	28/F	1
Waqas et al. [11]	Confusing ‎presentation of ‎Chaetomium brain ‎abscess	Case ‎report	‎28/F‎	1
Haider et al. [12]	Pre-operative voriconazole in patients undergoing surgery for central nervous system fungal infections: special report	Cohort study	‎36 ± 18‎	47; M, 34 (72.3%); ‎F, 13 (26.6%)
Altaf et al. [13]	Atypical radiological presentation of intracranial intra-axial fungal infection: a case report	Case report	20/F	1
Anis et al. [14]	A rare case of brain abscesses caused by Acremonium species	Case report	18/M	1
Wasay et al. [15]	Preoperative antifungal therapy may improve survival in patients with Aspergillus brain abscess	Cohort study	Preoperative, 51 ± 17 (7/4); postoperative 52 ± 20 (10/4)	25; M, 17 (68.0%); F, 8 (32.0%)
Mushtaq et al. [16]	Cerebral phaeohyphomycosis due to Rhinocladiella mackenziei in an immunocompetent patient: a case report and review of literature	Case report	42/M	1
Ijaz et al. [17]	Fungal neurological sequelae in a hyper immunoglobulin E syndrome from Peshawar, Pakistan: an unusual presentation	Case report	11/F	1
Waqas et al. [18]	Cerebral aspergillosis and pulmonary tuberculosis in a child with chronic granulomatous disease	Case report	4/M	1

Results

In total, 140 patients were included from nine case reports, one case series, and four cohort studies. The majority of cases were reported from Karachi/Sindh (113; 80.71%), while 22 (15.71%) were from Lahore/Punjab, two (1.42%) from Islamabad/Punjab, and 1 (0.71%) from Peshawar/Khyber Pakhtunkhwa (Table 3).

**Table 3:** Total number of patients with cerebral fungal infection

Study type	Number of studies	Total number of patients
Case report	9	9
Cohort study	4	129
Case series	1	2
Total	14	140

A total of 97 (69.28%) patients were males, and 43 (30.71%) were females. In cohort studies, the reported mean age ranged from 34.88 ± 19.49 to 52 ± 20, while patients described in case reports and case series were younger, with a mean age of 21.63 ± 10.66.

The most commonly reported presenting symptom was headache (58; 41.42%). Visual abnormalities, reported in 27 (19.28%), were the second most common symptom, followed by limb weakness (22; 15.71%) and seizures, accounting for 17 (12.14%) cases. Vomiting was reported in 16 (11.42%) of the patients, while fever, orbital swelling, and nasal blockade were reported in 7 (5%), 6 (4.28%), and 5 (3.57%), respectively (Table 4).

**Table 4:** Presenting symptoms of patients with central nervous system (CNS) fungal infections (N=140)

Presenting symptom	Number of patients (total, N=140)	Percentage occurrence
Headache	58	41.42%
Visual abnormalities	27	19.28%
Limb weakness	22	15.71%
Seizures	17	12.14%
Vomiting	16	11.42%
Fever	7	5%
Orbital swelling	6	4.28%
Nasal blockade	5	3.57%
Altered mental status	5	3.57%
Difficulty in speech	4	2.85%
Body rash	1	0.71%

On clinical examination, reduced limb power was observed in 5 (3.57%) patients, while reduced Glasgow Coma Scale (GCS) scores and a positive Babinski sign were reported in 2 (1.42%) patients each. Other findings included facial weakness in 1 (0.71%), diminished reflexes in 1 (0.71%), tachypnea in 1 (0.71%), and harsh vesicular breath sounds in 1 (0.71%) (Table 5).

**Table 5:** Clinical examination findings in patients with central nervous system (CNS) fungal infection (N=140)

Clinical examination findings	Number of patients (total, N=140)	Percentage occurrence
Reduced limb power	5	3.57%
Reduced Glasgow Coma Scale score	2	1.42%
Positive Babinski sign	2	1.42%
Facial weakness	1	0.71%
Diminished reflexes	1	0.71%
Tachypnea	1	0.71%
Harsh vesicular breath sounds	1	0.71%

Among the 140 reported cases, 67 patients (47.85%) were immunocompromised, whereas 20 patients (14.28%) were immunocompetent. Immune status was not specified in the remaining cases. In immunocompetent individuals, several predisposing factors were identified. Previous surgical intervention or device-related inoculation was reported in two patients (1.42%). Other factors included chronic paranasal sinusitis/respiratory infection in 2 (1.42%), history of trauma during imprisonment in 1 (0.71%), and localised facial swelling containing fungal spores in 1 (0.71%) (Table 6).

**Table 6:** Predisposing risk factors among immunocompetent patients with central nervous system (CNS) fungal infection (N=140)

Risk factors in immunocompetent patients	Number of patients (total, N=140)	Percentage occurrence
Previous surgical intervention or device-related inoculation	2	1.42%
Chronic paranasal sinusitis/respiratory infection	2	1.42%
Trauma during imprisonment	1	0.71%
Local swelling	1	0.71%

**Figure 1:**
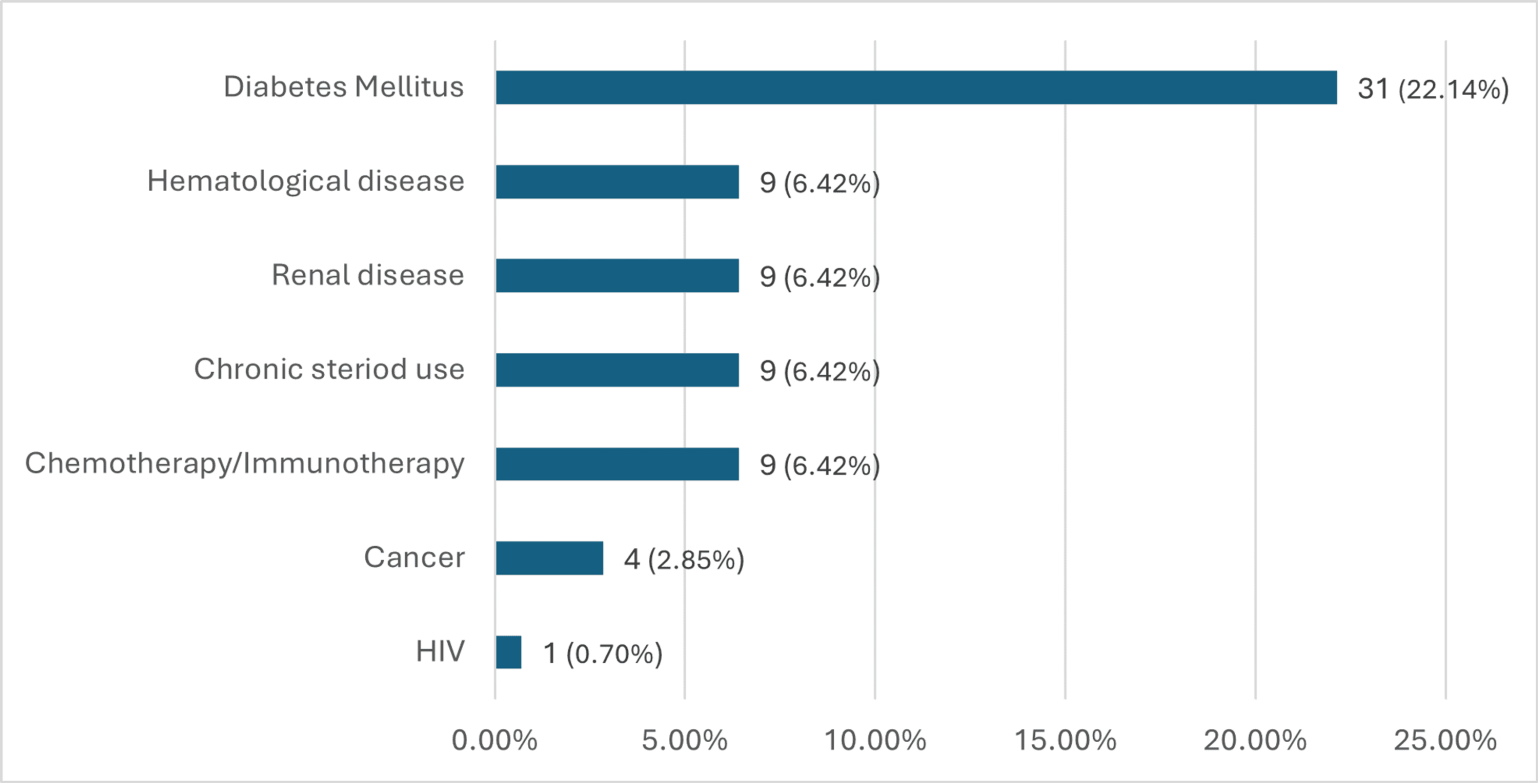
Past medical history contributing to immunocompromised state in patients with central nervous system (CNS) fungal infections (N=140)

Diabetes mellitus was present in 31 (22.14%) patients. Moreover, comorbidities such as hematological disease, renal disease, chronic steroid use, and chemotherapy were each present in 9 (6.42%) patients, while 67 (47.85%) patients had no prior contributing factors (Figure 1).

Biochemical investigations included baseline investigations - raised white blood cell count, ESR, and CRP. Other investigations included serum and cerebrospinal fluid (CSF) analysis for galactomannan and β-D-glucan, toxoplasmosis serology, and D-dimer tests.

Among all fungal infections, *Aspergillus *accounted for the most identified organism (46; 32.85%), followed by *Mucorales *(42; 30%), and *Madurella mycetomatis* (2; 1.42%) (Table 7).

**Table 7:** Distribution of fungal organisms identified among patients with central nervous system (CNS) fungal infections (N=140)

Most common organism identified	Number of patients (total, N=140)	Percentage occurrence
Aspergillus	46	32.85%
Mucorales	42	30.0%
Madurella mycetomatis	2	1.42%
Rhinocladiella mackenziei	2	1.42%
Cladophialophora bantiana	2	1.42%
Fonsecaea pedrosoi	2	1.42%
Chaetomium	2	1.42%
Acremonium	1	0.71%
Cryptococcus	1	0.71%

CT scan findings were variable, indicating an infiltrating mass, hypodense to iso-dense with surrounding oedema exerting mass effect. The frontal lobe was the most commonly involved region of the brain in 10 (7.14%) patients, followed by the temporal region in 8 (5.71%) (Table 8).

**Table 8:** Distribution of brain regions involved in central nervous system (CNS) fungal infections (N=140)

Most common brain region involved	Number of patients (total, N=140)	Percentage occurrence
Frontal	10	7.14%
Temporal	8	5.71%
Cerebellum	4	2.85%
Basal ganglia	2	1.42%
Fronto-parietal	2	1.42%
Thalamus	2	1.42%
Brain stem	2	1.42%
Parieto-occipital	1	0.71%
Parietal	1	0.71%
Occipital	1	0.71%

The majority of patients were treated with amphotericin B (82; 58.57%). Other antifungal drugs administered included voriconazole (40; 29.28%), itraconazole (31; 22.14%), and posaconazole (8; 5.71%). Patients may have received more than one antifungal medication; therefore, the sum of treatment counts exceeds the total number of patients (N=140), and percentages do not total 100% (Table 9).

**Table 9:** Medical treatment given in central nervous system (CNS) fungal infections (N=140)

Medical treatment	Number of patients (total, N=140)	Percentage occurrence
Amphotericin B	82	58.57%
Voriconazole	41	29.28%
Itraconazole	31	22.14%
Posaconazole	8	5.71%

Only 26 patients reported side effects of antifungal therapy. Among them, 23 (16.42%) had a complaint of acute kidney injury. Only 2 (1.42%) patients had visual symptoms, and 1 (0.71%) presented with a rash after the therapy.

The most common indication for neurosurgical management was worsening GCS score in 6 (4.28%) patients and progressive focal neurological symptoms in 2 (1.42%), while contrast-enhancing lesions on CT imaging were reported as the primary indication in 1 (0.17%) patient (Table 10).

**Table 10:** Indications for neurosurgical intervention in central nervous system (CNS) fungal Infection (N=140)

Indication of surgery	Number of patients (total, N=140)	Percentage occurrence
Worsening Glasgow Coma Scale (GCS) score	6	4.28%
Worsening focal neurological deficit	2	1.42%
Enhancing lesion on CT	1	0.17%

Among surgical approaches, neuronavigation-guided biopsy was the most frequently performed procedure in 29 (20.71%) patients, endoscopic debridement was performed in 16 (11.42%) patients, and subtotal resection in 15 (10.71%) patients. Other surgical procedures included excision of intracranial lesions in 10 (7.14%) patients, open radical surgery in 6 (4.28%) patients, and craniotomy in 6 (4.28%) patients (Table 11).

**Table 11:** Distribution of surgical treatment modalities in patients with central nervous system (CNS) fungal infection (N=140)

Surgical treatment	Number of patients (total, N=140)	Percentage occurrence
Neuronavigation-guided biopsy	29	20.71%
Endoscopic debridement	16	11.42%
Subtotal resection	15	10.71%
Excision of lesions	10	7.14%
Open radical surgery	6	4.28%
Craniotomy	6	4.28%
Debridement of local swelling on the skin	1	0.71%

A summary of the medically managed CNS fungal infection cases is detailed in Table 12. The majority of patients (113; 80.71%) underwent combined medical and surgical management. Within this group, clinical improvement was observed in 57 (50.44%) patients, and mortality occurred in 41 (36.28%) patients. While 26 patients (18.57%) underwent medical/conservative therapy, resulting in mortality in 17 (65.38%) and resolution in 2 (7.69%) patients (Table 13).

**Table 12:** Review of the literature documenting CNS fungal infections managed medically in the Pakistani population I: improved, LTFU: lost to follow-up, D: death, CSF: cerebrospinal fluid, MRI: magnetic resonance imaging, CT: computed tomography, ATT: anti-tuberculous treatment therapy

Author	Age/gender	Clinical presentation	Microbiological investigation	Radiological imaging	Brain area involved	Treatment given	Outcome
Mir et al. [9]	7/M	Left jaw swelling	Tissue culture: *Madurella mycetomatis*	CT - left parapharyngeal extension, CT - left infratemporal fossa extension, MRI - left inferior temporal lobe extension	Inferior temporal lobe	Amphotericin B, voriconazole	D
Tabassum et al. [19]	20/F	Raised red lesions on the head and neck, high-grade fever, dry cough, headache, intractable vomiting, weight loss, diplopia on left lateral gaze	CSF culture: *Cryptococcus neoformans*	MRI - nonspecific changes of increased signal intensity in the periventricular region	Periventricular area	ATT, amphotericin B - IV, fluconazole - oral	I
Arif et al. [20]	21/F	Headache, weight loss, low-grade fever	CSF: positive Cryptococcal antigen	MRI - bilateral lesions in centrum semiovale, periventricular white matter, internal capsule, and lentiform nucleus. Hypointense on T1W1, hyperintense on T2W1, bilateral mild dilation of the temporal horns of the lateral ventricles	Centrum semiovale, periventricular white matter, internal capsule, lentiform nucleus	ATT + high-dose steroid, amphotericin B and fluconazole	I
Faryal et al. [21]	30/M	Fever, headache, neck stiffness, and clear watery discharge from the nose	CSF culture: *Cryptococcus neoformans*	X-ray skull pneumocephalus		Ceftriaxone, metronidazole, no antifungal due to financial constraints	LTFU
Zulfiqar et al. [22]	40/M	Headache, fever, chills, vomiting, severe neck pain, photophobia, weight loss/weakness	CSF and blood culture: *Cryptococcus laurentti*	Brain MRI - T1W MRI: leptomeningeal enhancement and bilateral basal ganglia infarcts with cryptococcoma formation. T2W MRI: hyperintense areas in the right periventricular white matter and bilateral basal ganglia	Leptomeninges, basal ganglia, and periventricular white matter	IV liposomal amphotericin B, fluconazole	D
Ramzi et al. [23]	18/M	Fever and generalised, continuous headache associated with photophobia and phonophobia	CSF showed Cryptococcal species on India ink	CT scan: unremarkable	Meninges (meningitis)	IV amphotericin B	I

**Table 13:** Clinical outcomes in patients with central nervous system (CNS) fungal infection Percentages are calculated using the available number of patients (N) for each management category; therefore, the denominators differ between groups, and the totals do not sum to the overall study population.

Management	Outcome	No. of patients	Percentage occurrence
Medical/conservative (N=26)	Dead	17	65.38%
	Resolution/improvement	2	7.69%
	Worsening	-	-
	Recurrence	-	-
Medical + surgical (N=113)	Dead	41	36.28%
	Resolution/improvement	57	50.44%
	Worsening	2	1.76%
	Recurrence	1	0.88%

Discussion

CNS fungal infections are associated with high mortality; they usually spread from other organs through the hematological route [13]. This review demonstrates that CNS fungal infection presents with heterogeneous clinical and radiological patterns and affects both immunocompromised and immunocompetent individuals. It is also highlighted that cerebral fungal infections frequently require combined neurosurgical and antifungal management. In this study, nearly 80% of patients were reported from Karachi/Sindh, likely due to strong tertiary referral and diagnostic facilities in the metropolitan centre. The male predominance of 97 (69.28%) in our study aligns with a retrospective review of 29 cases, which shows a male-to-female ratio of 4.8:1 [24].

Clinical Presentation

Central nervous system fungal infection can manifest in various syndromes, including abscess, granuloma, meningitis, hydrocephalus, stroke, and myelopathies [25]. In our study, headaches (58; 41.42%), visual abnormalities (27; 19.28%), and focal neurological deficit/limb weakness (22; 15.71%) were the most frequent presenting symptoms (Table 4), although these symptoms were largely nonspecific. These findings are consistent with a study by Riddell and Wheat, which also identified headache, altered mental status, and focal neurological deficit as common presenting symptoms [26]. Such a deficit reflects the space-occupying nature of cerebral fungal abscesses, leading to mass effect [25].

The average duration of symptoms in our study was 24.7 days for headache, 8 days for fever, and 3 days for limb weakness. This variable timeline suggests that most patients sought medical attention only after the development of focal neurological deficits, while the preceding course was predominantly subacute. Most intracranial pathologies, such as tuberculous infection and pyogenic brain abscess, may produce similar constitutional findings, making differentiation challenging on clinical and radiological grounds [8]. This subacute presenting pattern signifies that CNS fungal infection frequently mimics other chronic intracranial pathologies that are endemic in the region, such as tuberculosis.

Similar diagnostic challenges were reported by Ali et al. in their study, where patients were initially treated with antitubercular therapy for presumed tuberculous meningitis, and in the study by Bhatti et al., where limb weakness was treated as Guillain-Barré syndrome [7,8]. In both reports, delayed recognition resulted in clinical deterioration, necessitating surgical intervention. Overall, the nonspecific symptom profile underscores the importance of maintaining a high index of suspicion for cerebral fungal infection, particularly in the presence of relevant risk factors, to enable timely diagnosis and management.

Pathogenesis

Recent understanding of the human mycobiome provides significant insights regarding fungal invasion of the central nervous system. Fungi normally colonise the gastrointestinal, respiratory, skin, and genitourinary system, where it continuously interacts with the host immune system [27]. Recognition of fungal pathogens is mediated through pattern-recognition receptors, particularly dectin-1 and Toll-like receptors (TLRs), which identify fungal cell wall components and initiate protective immune response. Dectin-1 specifically recognises β-1,3 glucans within fungal cell walls and acts synergistically with TLR2 to promote cytokine production, phagocytosis, and fungal clearance [27]. Disruption of this balanced microbiome by diabetes, antibiotics, or chronic critical illness can facilitate fungal overgrowth and translocation into the bloodstream, leading to cerebral seeding [27]. Moreover, low immunity also increases the permeability of the blood-brain barrier, which facilitates penetration of fungi into the brain [28]. Following CNS invasion, fungal organisms, including *Aspergillus *and *Mucorales*, can infiltrate vascular structures, resulting in thrombosis, cerebral infarction, and extensive disease progression, all of which contribute to poor clinical outcomes [28,29]. This mechanism may explain our findings, where the majority of patients were immunocompromised due to diabetes (Figure 1) or underwent an antecedent procedure and had sinus disease, suggesting breach of the mucosal barrier rather than primary neurotropism, and showed high mortality.

Predominant Fungi

Our study highlights the predominance of *Aspergillus *(46; 32.85%) and *Mucorales *(42; 30%) (Table 7). This distribution aligned with other studies in the region, such as India, where Sundaram et al. reported *Aspergillus *(56%) and *Mucorales *(30%) as being the most common causes of CNS fungal infections [29]. *Aspergillus *species are commonly found in soil, decaying vegetables, and organic debris. Therefore, agricultural workers spending time in the field are more prone to its exposure and paranasal sinus infection, which can lead to cerebral extension [29]. Furthermore, the high prevalence of mucormycosis observed in our review may be attributed to the substantial burden of diabetes mellitus in Pakistan, which was the most frequently identified predisposing factor. A systematic review by Jeong et al. also highlights diabetes as the most common underlying condition associated with mucormycosis in 40% of patients [30].

Specific fungal taxa are often associated with distinct clinical syndromes. As reported by Sundaram et al., *Aspergillus *was predominantly associated with focal intracranial lesions, including granulomas and cerebral abscess [29]. On the other hand, the *Cryptococcus *species observed in our cohort predominantly presented with clinical features of meningitis. This potentially highlights that recognition of these organism-specific presentations may facilitate earlier diagnostic suspicion and guide empirical antifungal therapy.

Other fungal species included in our study were *Cladophialophora bantiana*, *Rhinocladirlla mackenziei*, *Chaetomium*, *Acremonium*, and *Madurella mycetomatis*. The identification of rare fungi in this review mirrors recent global trends reported by Lino et al., who highlighted an expanding diversity of a few other rare fungal pathogens capable of CNS invasion, including *Candida auris*, *Trichosporon *spp., *Blastomyces *spp., *Sporothrix *spp., *Talaromyces marneffei*, *Lomentospora prolificans*, and *Scedosporium *spp. [2]. These findings highlight the evolving epidemiology of fungal CNS infection, particularly in immunocompromised individuals.

Radiological Findings

Gavito-Higuera et al. reported that the causative agent of brain abscess cannot be reliably distinguished on routine MRI [31]. However, comparative analysis of fungal versus pyogenic abscesses has demonstrated that fungal abscesses are usually multiple and appear as a hypointense lesion on T1WI and a hyperintense core on T2WI with a surrounding rim of hypointensity. Furthermore, diffusion-weighted imaging (DWI) may demonstrate restricted diffusion within the abscess wall and in the intracavity projections while sparing the central core, a feature that may aid differentiation from pyogenic abscesses [31]. However, in this study, the findings remain mainly heterogeneous, with only two cases documenting hyperintense lesions on T2WI, while the majority of the patients showed multiple abscesses. In addition, magnetic resonance spectroscopy may provide additional diagnostic information by demonstrating metabolite levels such as lipids (1.2-1.3 ppm), lactate (1.3 ppm), alanine (1.5 ppm), acetate (1.9 ppm), succinate (2.4 ppm), choline (3.2 ppm) and fungal disaccharide trehalose (3.6 ppm), which has been discribed as a relatively distinctive marker for fungal infection [31].

The most common brain region involved was the frontal lobe, seen in 10 (7.14%) patients, followed by the temporal lobe, in 8 (5.71%) (Table 8). This predominance aligns with the literature that highlights that frontal lobe involvement is largely due to anatomical proximity to the anterior skull base, which facilitates intracranial extension of fungi from sinus disease [32]. The literature also highlights that the fungal abscesses may involve the basal ganglia, whereas pyogenic abscesses tend to spare the basal ganglia [31].

Biochemical Investigations

In our review, definitive diagnosis of cerebral fungal infection was most commonly established through histopathology and cultures obtained by stereotactic biopsy specimens. Histopathology using periodic acid-Schiff (PAS) and Grocott's methenamine silver (GMS) remains the diagnostic gold standard because it allows direct visualisation of fungal elements and provides definitive organism identification when combined with the culture [33]. However, culture results often require several days to weeks, creating significant diagnostic challenges in patients with rapidly progressive disease. This delay poses a significant clinical challenge where early treatment may be critical for preventing neurological deterioration [2]. Mohanty et al. reported that KOH mount examination enables rapid identification of fungal organisms and may facilitate earlier initiation of empirical antifungal therapy [34]. A similar approach is seen in a case report by Mushtaq et al., where empirical antifungal therapy was initiated on positive KOH staining, before culture confirmation was available [16].

For *Cryptococcus *infections, detection of cryptococcal antigen (CrAg) in cerebrospinal fluid provides a highly sensitive diagnostic modality and is particularly valuable in resource-limited settings, where advanced microbiological testing may be unavailable [35]. Similarly, serological markers like galactomannan and β-D-glucan can support the diagnosis of invasive fungal infection; however, their variable specificity and sensitivity limit their use as standalone diagnostic tools [36]. Overall, these findings highlight an important clinical dilemma in CNS fungal infections where initiation of empirical antifungal therapy is often necessary before gold-standard results are available, due to the aggressive nature of the disease.

Treatment Strategies

In this study, the majority of patients (113; 80.71%) underwent both medical and surgical intervention, which led to improvement in 57 (50.44%) patients with combined treatment, while mortality remained high in patients managed with only conservative therapy (17; 65.38%). The most commonly used medical therapy included amphotericin B in 82 (58.57%), voriconazole in 41 (29.28%), and itraconazole in 31 (22.14%) (Table 9). These findings are in contrast with the review by Takoutsing et al., where the most common treatment modality was only medical therapy in 4,481 (51.6%), with fluconazole used in the majority of patients (1891; 21.8%) [37]. Furthermore, a retrospective study conducted by Khan et al. found that patients who received preoperative antifungal therapy (specifically, itraconazole) showed significantly better outcomes postoperatively, highlighting the use of preoperative antifungal therapy for better outcomes [10]. In our study, the majority of medically managed cases showed that *Cryptococcus *species were managed by amphotericin. The literature suggests that a combination with flucytosine has shown improved survival as compared to amphotericin B alone [38]. However, access to flucytosine remains limited globally; nearly 40% of the world’s population has no access to this antifungal therapy [39]. This limited availability is particularly problematic in low- and middle-income countries. Consequently, clinicians often rely on alternative regimes, such as amphotericin B monotherapy or amphotericin B combined with fluconazole, which may explain the difference in treatment outcomes compared with those reported in international studies.

In this study, surgical intervention was most commonly undertaken in patients demonstrating worsening GCS scores and focal neurological deficit, likely due to an increase in the size of the intracranial abscess as depicted by an enhancing lesion on MRI (Table 10). Among the most commonly opted surgical procedures were neuronavigation-guided biopsy (29; 20.71%), endoscopic debridement (16; 11.42%), and subtotal resection (15; 10.71%). Consistent with the previous literature, surgery played a dual role by facilitating definitive diagnosis through tissue acquisition while simultaneously reducing intracranial mass effect and fungal burden [40].

Despite the use of combined medical and surgical management, mortality remained high. This may be attributed to delayed diagnosis, advanced disease, and the aggressive angioinvasive nature of fungal pathogens such as *Aspergillus *and *Mucorales *[28,29]. The overall combined mortality (medical therapy and combined medical and surgical therapy) was 58 (41.42%). This high mortality can be attributed to fungal CNS infections resembling other etiologies, such as TB, pyogenic brain abscess, and neoplasms, keeping the index of suspicion for fungal CNS infection low, as well as delayed referrals from healthcare units with limited resources. Similar mortality rates have been reported by Naik et al. (36%), Schwartz et al. (46%), and Ikram et al. (44%), suggesting that substantial mortality remains a persistent challenge despite advances in neurosurgical techniques and antifungal therapy [6,41,42].

Limitations

This narrative review has several limitations. First, the evidence base consisted predominantly of case reports, case series, and retrospective studies, making the findings susceptible to reporting bias. Second, substantial missing and inconsistently reported data across studies restricted detailed analysis of several variables, including symptom duration, physical examination, immune status, radiological characteristics, treatment-related adverse effects, and long-term outcomes. Third, the majority of included patients originated from a tertiary care centre in Karachi, which may reflect referral patterns rather than the true geographic distribution of CNS fungal infection in Pakistan. Finally, the heterogeneity of study designs, fungal pathogens, treatment, and outcome approaches preclude formal comparative analyses.

Clinical recommendations

**Figure 2:**
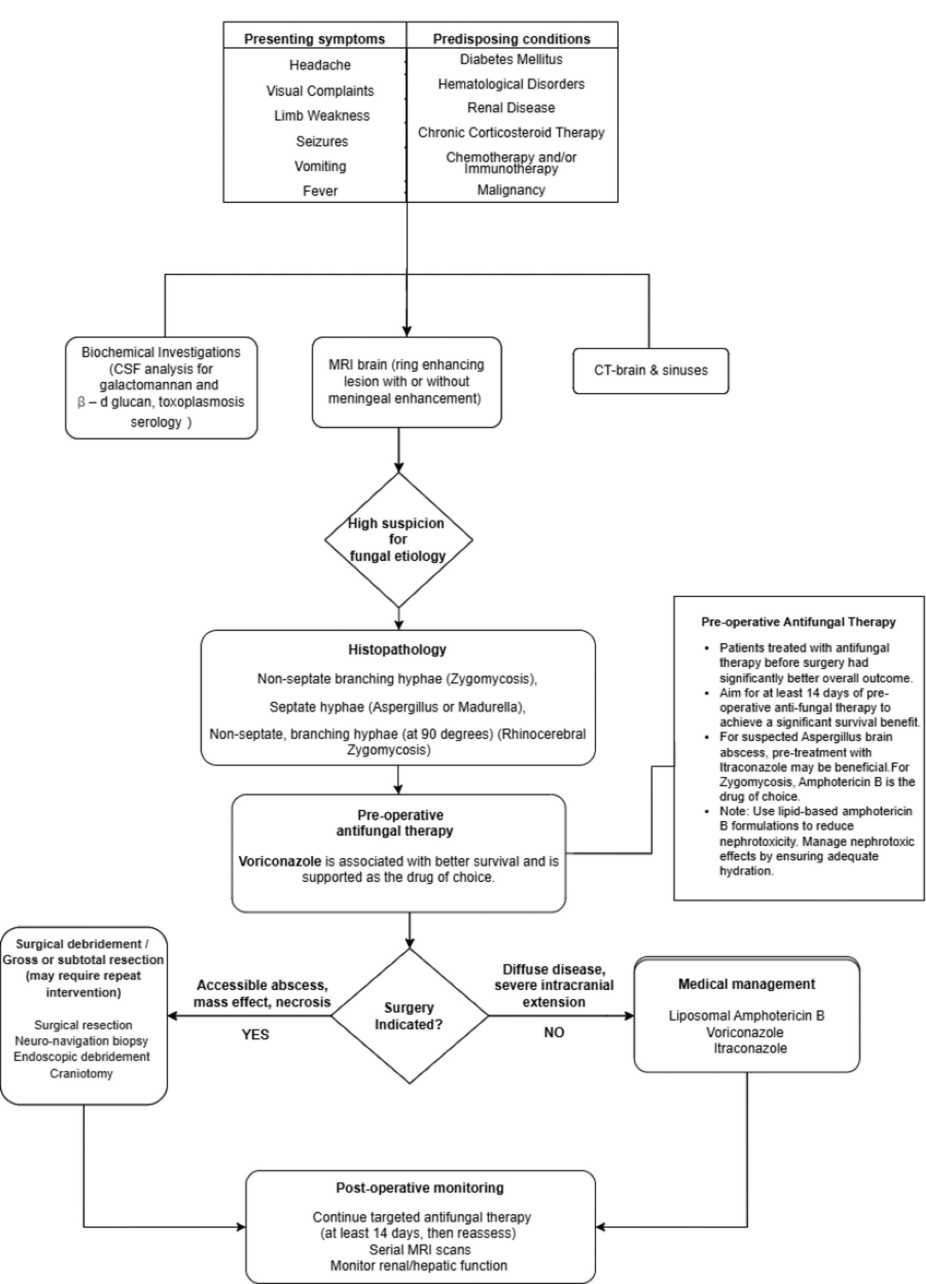
Author-proposed diagnostic and management algorithm for central nervous system (CNS) fungal infections

Clinicians should maintain a high index of suspicion for fungal infection in individuals presenting with neurological symptoms with co-existing diabetes, hematological malignancies, prolonged corticosteroid use, or a lack of clinical response to broad-spectrum antibiotics. Fungal etiology should also be considered in immunocompetent patients presenting with atypical findings (multiple lesions in the frontal lobe) and should be initiated on empirical antifungal therapy. Moreover, in the case of suspected cryptococcal CNS infections, prompt CSF evaluation and cryptococcal antigen testing should be performed where available. While treating and modifying underlying risk factors (improving glycemic control and immunosuppression), multidisciplinary management involving infectious disease specialists, neurosurgeons, and microbiologists is recommended to optimise diagnostic accuracy and outcomes. The authors of the study have proposed a diagnostic and managerial algorithm for CNS fungal infections in the local context (Figure 2).

## Conclusions

CNS fungal infections predominantly present with nonspecific clinical and radiological features. This review highlights the predominance of *Aspergillus*, *Mucorales*, and *Cryptococcus *in the Pakistani population. The majority of patients had diabetes as a potential predisposing risk factor. Histopathology and culture remain the diagnostic gold standard. Moreover, available reports suggest that combined medical and surgical management is likely beneficial in selected patients; however, the overall mortality remains high, and stronger evidence is needed to establish optimal treatment.
